# Automatic Identification System (AIS) Dynamic Data Integrity Monitoring and Trajectory Tracking Based on the Simultaneous Localization and Mapping (SLAM) Process Model

**DOI:** 10.3390/s21248430

**Published:** 2021-12-17

**Authors:** Krzysztof Jaskólski, Łukasz Marchel, Andrzej Felski, Marcin Jaskólski, Mariusz Specht

**Affiliations:** 1 Department of Navigation and Maritime Hydrography, Polish Naval Academy, ul. Śmidowicza 69, 81-127 Gdynia, Poland; l.marchel@amw.gdynia.pl (Ł.M.); a.felski@amw.gdynia.pl (A.F.); 2 Faculty of Electrical and Control Engineering, Gdańsk University of Technology, ul. Gabriela Narutowicza 11/12, 80-233 Gdansk, Poland; marcin.jaskolski@pg.edu.pl; 3 Department of Transport and Logistics, Gdynia Maritime University, Morska 81-87, 81-225 Gdynia, Poland; m.specht@wn.umg.edu.pl

**Keywords:** automatic identification system, reliability theory, integrity monitoring, trajectory tracking, extended Kalman filter

## Abstract

To enhance the safety of marine navigation, one needs to consider the involvement of the automatic identification system (AIS), an existing system designed for ship-to-ship and ship-to-shore communication. Previous research on the quality of AIS parameters revealed problems that the system experiences with sensor data exchange. In coastal areas, littoral AIS does not meet the expectations of operational continuity and system availability, and there are areas not covered by the system. Therefore, in this study, process models were designed to simulate the tracking of vessel trajectories, enabling system failure detection based on integrity monitoring. Three methods for system integrity monitoring, through hypotheses testing with regard to differences between model output and actual simulated vessel positions, were implemented, i.e., a Global Positioning System (GPS) ship position model, Dead Reckoning and RADAR Extended Kalman Filter (EKF)—Simultaneous localization and mapping (SLAM) based on distance and bearing to navigational aid. The designed process models were validated on simulated AIS dynamic data, i.e., in a simulated experiment in the area of Gdańsk Bay. The integrity of AIS information was determined using stochastic methods based on Markov chains. The research outcomes confirmed the usefulness of the proposed methods. The results of the research prove the high level (~99%) of integrity of the dynamic information of the AIS system for Dead Reckoning and the GPS process model, while the level of accuracy and integrity of the position varied depending on the distance to the navigation aid for the RADAR EKF-SLAM process model.

## 1. Introduction

Nowadays, due to increased maritime traffic, particular attention should be paid to collision avoidance. Technological solutions for safe navigation have forced the downsizing of ships’ crews. Using onboard navigation systems, it is possible to define the coordinates of one’s ship [1]. In relation to the other ships, one must rely on radio-navigational systems, e.g., the Automatic Identification System (AIS) [2]. Safe navigation based on reliable and accurate positioning is still an unsolved problem. Analyses of AIS data revealed the issue of numerous AIS messages containing incorrect coordinates [3]. Additionally, static and dynamic AIS data, received and recorded on a data carrier, are occasionally false or incomplete, as they contain empty fields. There are also cases of system synchronization failures, i.e., the inability to receive AIS position reports in a defined time interval or reception of only fragmentary data, not including heading (HDG) or rate of turn (ROT), or both. Moreover, reliability limitations may result from the incorrectly estimated course over ground (COG) or speed over ground (SOG), falsely describing the dynamics of the vessel.

Navigation system performance is measured through the following indicators [4,5]: accuracy, availability, continuity, and integrity [6]. The latter is critical for assessments of AIS usefulness in navigation. Originally invented as a radiobroadcast channel, AIS does not transmit any message on data integrity. Moreover, continuity of AIS operation is not guaranteed, which limits the system application in navigation down to the role of a supporting tool. Automatic radar plotting aid (ARPA) is the main tool for vessel monitoring and anticollision maneuvering.

To increase AIS reliability, it is necessary to develop methods of AIS integrity monitoring and simulated trajectory tracking. Therefore, the authors propose the use of Extended Kalman Filter (EKF) based on the distance and bearing to navigational aid (beacon), the GPS process as a simulation model, and the Dead Reckoning (DR) process as a simulation models to monitor AIS integrity. The model was developed to represent the motion of a vessel both in a straight-line and while maneuvering. Based on the differences in the positions estimated by the object simulation model, the AIS system can apply methods known from the reliability theory and determine the degree of system integrity characterized by the level of confidence in the information sent by AIS. The authors formulated the following research hypothesis: the estimation of coordinates using GPS, EKF with bearing and distance measurements and DR allows the reconstruction of the ship trajectory to verify the reliability of the AIS system through a stochastic process based on Markov chains.

Kalman filters have been widely used in mobile ship navigation and system integration. If the linearization procedure is conducted with the use of the Kalman filter, then an EKF is obtained [7]. Filtering algorithms operate in a discrete-time manner. Knowledge about the system dynamics and its correct modelling is the main issue in Kalman filter implementation [8]. In systems with nonlinear dynamics, the motion model equations are linearized using an EKF. Linearization is conducted using partial derivatives of nonlinear state functions or their Taylor series expansion [9].

Kalman filter and its extensions and mutations can be effective for predicting the trajectories of other ships, as demonstrated in [10], whereby an approach to AIS data integrity monitoring was based on hypotheses testing through chi-squared and the Generalized Likelihood Ratio (GLR). The Interacting Multiple Model (IMM) for state estimation of the Constant Velocity (CV) and the Constant Turn Rate Velocity (CTRV) model were used. The alternative to the EKF is the Unscented Kalman Filter (UKF), based on Unscented Transformation (UT) used for statistics computation (first two moments) of a random vector undergoing transformation by a nonlinear function. Another option is the particle filter (PF) that uses the Monte Carlo method, i.e., a numerical method allowing, e.g., the calculation of integrals which are impossible to solve analytically [11].

There are, however, other approaches, like applying artificial neural networks for tracking [12]. Also, an autonomous path planning model based on deep reinforcement learning (DRL) was proposed to realize intelligent path planning for a ship in an unknown environment [13]. A deep neural network (DNN) based on the DDTree model to predict waterway depth for ships combines a decision tree and DNN, and is trained and tested on the AIS and Global Mapper data. This model can provide an accurate prediction of waterway depth and compensate for the shortage of waterway depth monitoring means [14]. However, it should be remembered that artificial neural networks operate on fuzzy concepts and do not work in multistage calculations unless one uses a whole set of various neural networks which are not necessarily linearly connected. The output from one network goes to the input of another network, which operates in a single-step manner.

This paper presents the implementation of three process models: EKF-SLAM (Simultaneous Localization and Mapping), based on radar bearing and distance to navigational aid, and DR and GPS simulation of AIS data to monitor the integrity of AIS data. If a process of data transfer which provides a user with accurate navigational data at any moment is a reliable process, it can be viewed as the functioning of an information channel and can be assessed with methods from reliability theory. It is crucial to adapt the research tools and conduct reliability analyses in the simulated area and use a methodology that provides reliable research results. This was done through the development of research methods for AIS information availability and integrity based on homogeneous Markov chains.

To estimate the availability and integrity of AIS information, the following test methods were contemplated in previously published work:

a. Fault Tree Analysis (FTA)—the method of analysis designed to determine which type of unfitness, damage of the object, an external event or a combination thereof can generate an object failure. This method is presented in the form of a failure tree [15];
b. Multivariate statistical analysis—a method that examines the confidence degree of received information. The method is based on the analysis of recorded data detailing the study of information affecting the safety of navigation. It is possible to determine integrity and availability of binary channel transmission. Multivariate statistical estimation is used in this method [16];
c. Operating states graph—the method of presentation for the reliability structure of the object. This method is used for reliability evaluation. On the basis of stochastic processes, this method is an effective way for reliability estimation of renewable objects [15];
d. Stochastic methods with the use of Markov and semi-Markov process—provide a convenient mathematical apparatus enabling the description and investigation of actual random processes. They are an important class of stochastic processes, which allows a mathematical description of the change of random quantities in time [15,16];
e. Probability model based on the chi-square test presented in [10]. The chi-square test is used to test hypotheses. The value of the test is assessed using the chi-square distribution. The test most often used in practice. We can use it to test the compliance of both measurable and immeasurable features.
f. Generalized likelihood ratio (GLR) control method for detecting changes in the parameters of system for individual observations. The method usually used for monitoring system is based on taking a sample of *n* observations at each sampling time point, where *n* is large enough that a regression model can be fitted at each sampling point using these *n* observations [17,18].

This study is motivated by the constantly growing problem of spoofing and jamming GPS systems which has been observed over the recent years. This can lead to the absence of transmissions from ships, or transmissions of wrong data, i.e., data which are not coherent with the other data. One side of this problem involves the difficulties faced by ships’ watch officers, while the other side involves troubles in practices of services such as Vessel Traffic Services or Government Agencies like Coast Guard etc. Another problem, more common and known for many years, is the question of the integrity of the AIS data.

The first related area is the detection of anomalies in AIS data. The beginning of AIS (before 2014) was a period of poor quality AIS data, as, for example, noted in a study published by Harati-Mokhtari et al. [19]. That study indicated the inconsistencies of data, e.g., vague or incorrect AIS entries for destinations. Felski and Jaskolski [20] discussed the completeness and integrity of AIS data in collision avoidance with data from 2006 to 2007, and 2010 to 2012. The findings suggested a high level of dynamic data integrity of AIS. With additional satellites launched in 2014, the quality of AIS data has significantly improved. However, there is still a debate on the correctness of static data in AIS, as they are input manually. Lensu and Goerlandt [21] stated that the AIS stream is vulnerable and may contain considerable amounts of erroneous data. Therefore, it should be cross-checked against other information apart from just the checksum, which points to the idea of the elaboration of data verification algorithms. In that period, nobody was interested in taking into account GPS errors, which are the source of serious problems nowadays.

AIS data anomaly problems are essentially different from other anomaly detection problems known in the literature. For instance, anomalies in AIS data, such as the coordinates of a ship which has strayed from its route or into a restricted area, no rational speed of the ship, a ship which is traveling significantly above or below the standard speed, etc., can be easily noticed by operators. In this research, we are interested in no self-evidence errors, but possible disturbed analyses of the ship movement. This can be useful when analyzing the movement on the borders of the ranges of two AIS-base stations, when some gap area can exist or when the exact trajectory of a ship is important, for example, after a disturbance in the operation of the GPS, during investigations of collision accidents, etc. Similar issues characterize the process of analyzing data transmitted via satellite AIS, when gaps between received messages are in the order of hours [22].

Trajectory analyses with the AIS data include two aspects: trajectory extraction and trajectory prediction, usually based on the reported spatiotemporal sequence data [23,24]; however, these kinds of studies are directed toward logistics aspects and usually detect some specifics in a ship’s behavior or general data regarding the ship’s movements.

Alizadeh, et al. [25] proposed a method to predict a ship’s trajectory in 10–40 min based on its historical AIS data. For such use, there is an interesting idea of integrating AIS data with other data, as presented by Lensu and Goerlandt [21] for the Baltic Sea, especially for environmental safety.

The trajectory of a ship’s movement, determined on the basis of reliable dynamic data, supports the efficiency and safety of the tracked ship. Such a trajectory can be built on the basis of archival data from the position reports of the AIS system. The Artificial Neural Network (ANN) model can be used to predict ship movement along with smoothing the ship’s trajectory. The proposed framework smoothed noises in the raw AIS data by combining a Hampel Filter (HF) and a Butterworth Filter (BF) [26]. This is the latest research approach in the field of ship motion trajectory estimations.

To the best of the authors’ knowledge, the combination of methods proposed in this paper has never been used in AIS data integrity monitoring and simulation trajectory tracking to detect inaccuracies in dynamic data exchanges.

The following structure was adopted for the remaining part of the paper. Section 2 presents the AIS data simulation for the needs of this work, the design of the filter, motion model and measurement model with an integrity architecture model. The research outcomes are given in Section 3 and discussed in Section 4. Finally, conclusions from the research outcomes of tracking data integrity examination are drawn in Section 5.

## 2. Materials and Methods

### 2.1. Simulation of Automatic Identification System (AIS) Dynamic Data

Each vessel equipped with a Class A AIS receiver transmits a position report in accordance with the technical specification ITU-R M.1371 [27]. Position reports contain dynamic data, inter alia: ROT, SOG, Longitude, Latitude, COG. The reporting interval between two consecutive AIS position reports received from the same vessel equipped with AIS Class A receiver are from 2 to 180 s, and were presented in [27].

The AIS transponder receives data from connected sensors; among other things, these are HDG and ROT. The measured parameters come from gyro-compasses, whereas geographical coordinates, i.e., latitude and longitude, as well as SOG and COG, come from the positioning system receiver. To model a ship’s motion, the latitude, longitude, SOG, COG, HDG and ROT are fundamental components.

In the conducted experiment consisting of simulating a ship’s movement, the object-ship generates positions according to the interval provided for the AIS class A system in three variants:

- based on EKF-SLAM estimator, on the basis of radar bearing and distance to fixed navigation aid;
- based on the mathematical count of the ship’s movement (Dead Reckoning);
- based on the simulated position of the object using the GPS system.

**Figure 1:**
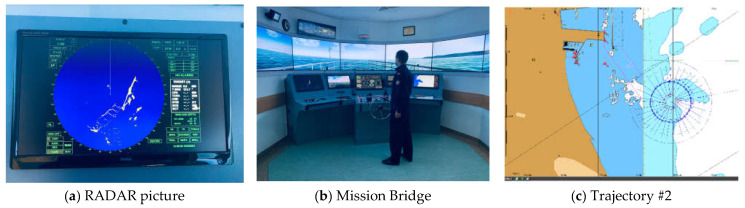
Simulation of the ship’s movement in accordance with the assumptions made in the Navigational and Maneuvering Simulator Group of the Polish Naval Academy. (**a**) RADAR picture of the simulated trajectory, (**b**) mission bridge (**c**) simulated trajectory # 2. Photo: Ł. Marchel.

The simulation was conducted in the Navigational and Maneuvering Simulator Group of the Polish Naval Academy in Gdynia. The simulation area was Gdańsk Bay (Figure 1).

In view of the above, three ship motion models were built to develop the AIS dynamic data integrity model, descriptions of which are presented in Section 2.3.

### 2.2. Trajectory Tracking Process Models

This section aims to present the concept of three process models providing a data position monitoring service of a vessel trajectory. GPS, DR methodology and RADAR EKF-SLAM filter output were employed to compare estimated data with a simulation motion model to find instances of position inaccuracy [28]. The two-state integrity model based on Markov chains was used to assess the integrity of AIS data

It was assumed that the vessel would navigate along a simulated trajectory. In the time interval Δ*t* = *t*(*k*) − *t*(*k* − 1), at time step *k*, the vessel equipped with an AIS receiver delivers position reports with dynamic data, i.e., course over ground *COG*(*k*), speed over ground *SOG*(*k*), rate of turn *ROT*(*k*) and geographical coordinates (*φ*(*k*), *λ*(*k*)).

Cartesian coordinates *xs*(*k*), *ys*(*k*) were converted from geographical coordinates (*φ*(*k*), *λ*(*k*)).

Based on the measurements obtained at each time *k*, the coordinates were estimated in the time interval Δ*t* using the following estimation framework: *xs*(*k*), *ys*(*k*), *COG*(*k*), *SOG*(*k*), and *ROT*(*k*).

Cartesian coordinates constituted the initial dataset for the simulated ship motion, GPS, DR and the RADAR-EKF-SLAM. The measurement vector *z*(*k*) serving as the filter input was computed on the basis of the following estimation framework: *xs*(*k*), *ys*(*k*), x(i), y(i).

Where x(i) and y(i) are navigation aid coordinates.

#### 2.2.1. Dead Reckoning (DR)

Based on the simulation assumptions, DR—motion model was defined as follows:

(1) $$\left(k\right)=\left[\begin{array}{c} X\left(k\right)\\ Y\left(k\right)\\ COG\left(k\right) \end{array}\right]=\left[\begin{array}{c} X\left(k-1\right)+SOG\left(k\right)\cdot \Delta t\cdot \cos\left(COG\left(k-1\right)\right)\\ Y\left(k-1\right)+SOG\left(k\right)\cdot \Delta t\cdot \sin(COG\left(k-1\right)\\ COG\left(k-1\right)+ROT\left(k\right)\cdot \Delta t \end{array}\right]$$

where:

ε(k)—state vector of the DR motion model,

*X, Y*—coordinates on the *X and Y* planes,

*K—*moment of time,

*X*(*k*) and *Y*(*k*)—simulated Cartesian position coordinates of the vessel at moment *k*,

∆*t*—the time from the moment *k* − 1 to moment *k*,

*SOG*(*k*)—vessel velocity at moment *k*, and

*COG*(*k*)—course over ground at moment *k*.

#### 2.2.2. RADAR Extended Kalman Filter (EKF) Simultaneous Localization and Mapping (SLAM)

The Kalman Filter, based on an iterative prediction-correction process implements the Bayesian framework [10]. A linear process is used to model the AIS system dynamics, while the KF optimizes state estimation. The estimated value vector ξ(*k*) represents the state of the system at the moment *k*. The KF is a convenient method of estimating the values of linear variables. However, KF does not meet the requirements of estimating the values of non-linear variables. In marine applications, where the parameters used in the estimation process are non-linear and the measurement data are not highly noisy in the case of large vessels, the use of the Extended Kalman Filter (EKF) has proven to be a better solution [10]. In Section 2.2.3, the model for vessel motion at sea was introduced, which constitute an EKF-based estimation framework. The Extended Kalman Filter was defined as [29] given we know ((k),P(k−1),u(k),z(k)):

(2) $$\bar{\xi }\left(k\right)=g\left(\left(k-1\right),u\left(k\right)\right),$$

(3) $$\bar{P}\left(k\right)=\left(G\left(k\right)\cdot P\left(k-1\right)\cdot G^{T}\left(k\right)+R\left(k\right)\right),$$

(4) $$K\left(k\right)=\bar{P}\left(k\right)\cdot H^{T}\left(k\right)\cdot {\left(H\left(k\right)\cdot \bar{P}\left(k\right)\cdot H^{T}\left(k\right)+Q\left(k\right)\right)}^{-1},$$

(5) $$\left(k\right)=\bar{\xi }\left(k\right)+K\left(k\right)\cdot (z\left(k\right)-h\left(\bar{}\left(k\right)\right),$$

(6) $$P\left(k\right)=\left(I-K\left(k\right)\cdot H\left(k\right)\right)\cdot \;\bar{P}\left(k\right),$$

return ξ(k), P(k).

The measurement model was defined as:

(7) z(k)=h(ξ(k))+(k),

where:

$\bar{\xi }\left(k\right)$—estimated state vector for the time moment *k*,

*g*(∙)—non-linear, multivariate function modelling the system in the state *ξ*(*k* + 1),

*z*(*k*)—measurement vector for moment *k*,

*h*(∙)—nonlinear function that relates the measurement *z*(*k*) to the state *ξ*(*k* + 1),

*u*(*k*)—vector of process disturbances, the additive noise in the process,

*ε*(*k*)—vector of measurement errors, measurement domain,

G(k)—the matrix of the process model. Jacobian matrix of the function *g*(·),

*I*—identity matrix,

$\bar{P}\left(k\right)$—covariance matrix of prediction errors,

*H*(*k*)—the matrix of the measurement model at time *t*(*k*), Jacobian matrix of the function *h*[*ξ*(*k*)],

*K*(*k*)—Kalman Gain,

$\bar{P}\left(k\right)$—covariance matrix of filtration errors,

*Q*(*k*)—the covariance matrix of the measurement model, and

R(k)—the covariance matrix of the motion model errors.

Determining the position with the EKF-SLAM estimator requires knowledge of the object’s position and spatial orientation based on the positioned navigation aids. The test assumes that the ship’s motion will be simulated in a two-dimensional plane in a local frame of reference. The position of the navigation aid is simulated by a computer program. The vector of the position of the navigation aid takes the form:

(8) $$M\left(k\right)={\left[X\left(1\right),Y\left(1\right)\right]}^{T}$$

where:

M(k)—vector of the position of the navigation aid (beacon),

X(1),Y(1)—the coordinates of the navigation aid (beacon).

The presented variant assumes the presence of only one navigation aid. The vessel is moving at a constant speed, i.e., also a constant turning speed when changing course.

The extended Kalman filter is divided into three phases. The first is the filter initiation phase. In the ship motion simulation developed for the purposes of this article, the first phase takes place at the beginning of the study, after the object’s position has been changed based on the bearing and the distance to the navigation aid [29].

The matrices *G*(*k*) and *H*(*k*) were obtained as the Jacobians of *g*(∙) and *h*(∙) using Equations (9) and (10) [29]:

(9) $$\begin{array}{c} G\left(k\right)=\frac{\partial g}{\partial \xi }|\bar{\xi }\left(k-1\right)|\left(k-1\right),u\left(k\right)=\\ \begin{array}{ccccc} 1 & 0 & -\sin COG\left(k-1\right)\Delta t\cdot SOG & 0 & 0\\ 0 & 1 & \cos COG\left(k-1\right)\Delta t\cdot SOG & 0 & 0\\ 0 & 0 & 1 & 0 & 0\\ 0 & 0 & 0 & 1 & 0\\ 0 & 0 & 0 & 0 & 1 \end{array} \end{array}$$

(10) $$H\left(k\right)=\frac{\partial h}{\partial \xi }|\bar{\xi }\left(k\right)|\left(k-1\right)=\left[\begin{array}{cc} \frac{\Delta X}{r} & \begin{array}{cc} \frac{\Delta Y}{r} & \begin{array}{ccc} 0 & -\frac{\Delta X}{r} & -\frac{\Delta Y}{r} \end{array} \end{array}\\ -\frac{\Delta Y}{r^{2}} & \begin{array}{cc} \frac{\Delta X}{r^{2}} & \begin{array}{ccc} 1 & \frac{\Delta X}{r^{2}} & -\frac{\Delta X}{r^{2}} \end{array} \end{array} \end{array}\right],$$

where:

*g*(∙)—non-linear, multivariate function modelling the system in the state,

*h*(∙)—non-linear function that relates the measurement *z*(*k*) to the state, and

*r*—distance between ship and navigational aid (beacon).

The matrix R(k) causes the inclusion of additional uncertainty caused by noisy, error-prone indications of the value of assumed motion control *u*(*k*) into the system filter and is defined as:

(11) $$R\left(k\right)=W\left(k\right)\cdot M\left(k\right)\cdot W{\left(k\right)}^{T},$$

Designation of the matrix R(k) requires the calculation of Jacobian *g*(∙), with respect to noisy control input [29].

(12) $$\begin{array}{c} W\left(k\right)=\frac{\partial g}{\partial \xi }|\bar{\xi }\left(k\right)|\left(k-1\right),\varepsilon \left(k\right)=\\ \begin{array}{cc} \Delta t\cdot \cos COG\left(k\right) & -SOG\cdot \Delta t\cdot \sin COG\left(k\right)\\ \Delta t\cdot \sin COG\left(k\right) & SOG\cdot \Delta t\cdot \sin COG\left(k\right)\\ 0 & 1\\ 0 & 0\\ 0 & 0 \end{array} \end{array}$$

The mean error of the distance and bearing measurements, as well as the ship speed, are determined at each course change in matrices *M* and *Q*.

(13) $$Q=\left[\begin{array}{cc} \sigma_{r}^{2} & 0\\ 0 & \sigma_{\beta }^{2} \end{array}\right],$$

(14) $$M=\left[\begin{array}{cc} \sigma_{v}^{2} & 0\\ 0 & \sigma_{ROT}^{2} \end{array}\right].$$

where:

$\sigma_{r}^{2}$—mean error of distance measurement to the navigational aid;

$\sigma_{\beta }^{2}$—mean error of the bearing measurement to the navigational aid (beacon);

$\sigma_{v}^{2}$—mean error of speed over ground (SOG) measurement;

$\sigma_{ROT}^{2}$—mean error of Rate of Turn (ROT) measurement.

The next step in the EKF filter is the correction phase. The filtration algorithm incorporates a set of randomly erred measurements z(k) into the observed navigational aid.

#### 2.2.3. Motion Model

The object’s initial position in the local coordinate system was assumed:

xs(k)=0, ys(k)=0. The error value for the start position is:

(15) $$\sigma_{Xs0}=0,$$

(16) $$\sigma_{Ys0}=0.$$

In the following model, the initial state was defined as follows:

(17) $$x\left(0\right)={\left[Xs\left(k\right),\;\;Ys\left(k\right),\;\;COG\left(0\right),\;\;r\left(0\right),\;\;\beta (0)\right]}^{T},$$

with Xs(k), Ys(k) as position estimates in the Cartesian plane, *COG*(0), range r(0) and bearing β(0).

Increment values COG(k), ROT(k) and SOG(k) have a random error in the normal distribution σ(COG), σ(ROT), σ(SOG). The distance and bearing to the navigation aid (beacon) in the filter initiation phase is 0.

(18) $$\Delta COG\left(k\right)\;\epsilon \;N\left(0,\sigma^{2}COG\right),$$

(19) $$\Delta SOG\left(k\right)\;\epsilon \;N\left(0,\sigma^{2}SOG\right),$$

(20) $$\Delta ROT\left(k\right)\;\epsilon \;N\left(0,\sigma^{2}ROT\right).$$

#### 2.2.4. Measurement Model

The measurement model requires the estimation of the coordinates of the position of the observed navigation aid. A series of measurements are required to determine position *j* of the aid. In this case, we specify the distance *z*(*r*) and bearing *z*(*β*) to the navigation aid. It was assumed that we would treat both the ship and the navigational aid as a point. The measurements are burdened with a random error Δz(r), Δz(β) in a normal distribution with the standard deviation previously determined for the radar: σ(r) for measured distance, σ(β) for measured bearing.

Bearing this in mind, the measurement model to i of navigational aid (beacon) is defined by:

(21) $$h\left(i,\bar{x}\left(k\right)\right)=\left[\begin{array}{c} z\left(r\right)\\ z\left(\beta \right) \end{array}\right]=\left[\begin{array}{c} \sqrt{{\left[(x\left(i\right)-xs\left(k\right)\right]}^{2}+{\left[(y\left(i\right)-ys\left(k\right)\right]}^{2}}\\ tan^{-1}\left[\frac{y\left(i\right)-ys\left(k\right)}{x\left(i\right)-xs\left(k\right)}\right] \end{array}\right],$$

and:

(22) $$\Delta z\left(r\right)\epsilon \left(0,\;\sigma_{r}^{2}\right),$$

(23) $$\Delta z\left(\beta \right)\epsilon \left(0,\;\sigma_{\beta }^{2}\right),$$

where:

δ(r) = 1% the radar distance from the navigation aid (beacon) to the maneuvering object, and

δ(β) was arbitrarily set at 0.3 deg.

The EKF-SLAM, DR, GPS models are used in the same scenarios of vessel movements. The EKF-SLAM model is based on the radar measurements at time instance *k*, with position estimates in the Cartesian planes. The Dead Reckoning model, which is far less computationally heavy, is based on the kinematic formula model and should reflect a vessel motion in a nearly straight line. GPS, EKF-SLAM and DR were applied in five measurement campaigns with different vessel dynamics. As a result of EKF-SLAM and DR computation, one obtains the integrity measure, which indicates whether an AIS user can rely on a position report or not. Initial state vector *x*(0), described by position, COG, distance r, bearing β to navigational aid (beacon) was set according to:

(24) $$\xi (k)={\left[\begin{array}{ccc} Xs\left(k\right) & Ys\left(k\right) & \begin{array}{ccc} COG\left(k\right) & r & \beta \end{array} \end{array}\right]}^{T}.$$

#### 2.2.5. Integrity Model

In addition to the modelling of vessel trajectory tracking, the main goal of this study is to deliver a measure of the AIS data integrity. Integrity includes the ability of the system to provide timely warnings to users when the system should not be used for navigation.

The operating time of the information delivered via AIS is characterized by the exponential distributions of the lifetime and the time of failures. If a process of data transfer, resulting in providing a user with complete data at any moment, is a reliable process, it can be viewed as the functioning of an information channel and can be investigated with methods included in the theory of reliability. In connection with the above, a model based on the Theory of the Markov Processes related to Operating Technical Objects was proposed. It is known that the Markov chain is characterized by the fact that the state at time *n* + 1 depends only on state at the time *n*, and is independent of the state in the preceding moments.

Based on the above assumption, for incoming data, a stochastic probability matrix of transitions between integrity states of AIS was determined, enabling the identification of the source of unreliable data. The stochastic matrix presents the intensity of transitions between states.

The time value of the state of system availability is a value that can be described using stochastic processes. Therefore, the authors developed a model of AIS integrity using the Markov chains methodology.

The stochastic process was denoted by the symbol:

(25) {S(t):t∈T},

Special case of a stochastic process is a random sequence {*S*(*n*):*n* ϵ (0, 1, 2, 3, …, *N*)} which is called a random chain. The values of random variables {*S*(*n*):*n* = (0, 1, 2, 3, …, *N*)} represent states of AIS integrity in Trajectory. Nature of state changes can be assumed as a Markov chain on a set of states:

(26) $$S=\left\{S_{1},S_{2}\right\},$$

where:

*S*_1_—State *S*(*t*) = 0 means that in moment *t*, the accuracy is more than $3M_{xy}^{}$, the system is in a failure state.

*S*_2_—State *S*(*t*) = 1 means that in moment *t*, the accuracy is less than $3M_{xy}^{}$, the system is in an operating state.

*N*—number of samples.

It was assumed that the intensity of the transition between states is dependent on the value of $M_{XY}^{}$, i.e., root mean square (RMS) error.

Where:

(27) $$M_{XY}^{}=\sqrt{m_{X}^{2}+m_{Y}^{2}},$$

and:

$m_{X}^{2}$ $m_{Y}^{2}$—distance between reference and RADAR, GPS or DR positions, *X* and *Y* planes,

$M_{XY}^{}$—root mean square error according to motion model.

The moments when the system is in the failure state are the moments of the navigational structure renewal.

The Markov chain is defined if the initial distribution:

(28) $$P=\left[p_{ij}:i,j\in S\right],$$

and matrix of transition probabilities:

(29) $$P\left(\Phi_{0}=i\right)=p_{i},i\in S,$$

where:

(30) $$p_{ij}=P\left(\Phi_{n+1}=j\;|\Phi_{n}=i\right),\;\;\;n=0,\;1,\;2,\;3,\ldots N,$$

were given.

Therefore, the stochastic matrix defines the probability of transitions between states of the integrity of AIS. The stochastic matrix presents the intensity of transitions between states.

Therefore, *p_ij_* means the probability of transition from state *i* ϵ *S* to state *j* ϵ *S* at *n* + 1.

In our case, the transition matrix takes the form of:

(31) $$P=\left[\begin{array}{cc} p_{00} & p_{01}\\ p_{10} & p_{11} \end{array}\right],$$

In addition, the initial distribution *p*(0) = [1, 0] was adopted for the study. This means that the system is in failure state *S*_1_.

An important role in the study of the processes described by Markov chains is played by boundary characteristics, especially the limits of probabilities $p_{i}\left(n\right)\;\mathrm{and}\;p_{ij}\left(n\right)\;\mathrm{at}\;n\to \infty .$ They describe the probabilistic behavior of the process over a long period of time.

The limit probability was determined from the dependence:

(32) $$\underset{n\to \infty }{\lim}p_{ij}\left(n\right)=\underset{n\to \infty }{\lim}P(\Phi_{n+1}=j|\Phi_{n}=i)=\underset{n\to \infty }{\lim}P(\Phi_{n+1}=j)=\pi_{j},$$

where:

$\underset{n\to \infty }{\lim}p_{ij}\left(n\right)$—limit probability of the transition from state *i* to state *j*.

$\underset{n\to \infty }{\lim}P(\Phi_{n+1}=j|\Phi_{n}=i)$—boundary value of the conditional probability that for any i,j∈S the process state at time n+1 depends only on the state at time n and does not depend on the states at earlier times

$\pi_{j}$—limit probability of an object being in state j∈S.

To reach the intended goal, it was necessary to solve the set of linear equations:

(33) $$\sum_{i\in S}\pi_{i}p_{ij}=\pi_{j},j\in S,$$

where:

$p_{ij}$—probability of the transition from state i∈S to state j∈S at time *n* + 1.

and

(34) $$\sum_{i\in S}\pi_{i}=1,$$

It means that the sum of the limit probabilities of the object being in state i is equal to 1.

Therefore:

(35) $$p_{00}+p_{01}=1,$$

and:

(36) $$p_{10}+p_{11}=1,$$

where:

$p_{ij}$—the probability of the intensity of the transitions between states i,j or remaining in state i,

*π*—limit probability.

Probabilities determine the stationary distribution of homogenous Markov chain π = [π_0_, π_1_] with the matrix of transition probabilities:

(37) $$P=p_{ij}:i,j\in S,$$

Limit probabilities were calculated by solving a set of equations based on the matrix product:

(38) $$\left[\begin{array}{cc} \pi_{0} & \pi_{1} \end{array}\right]\left[\begin{array}{cc} p_{00} & p_{01}\\ p_{10} & p_{11} \end{array}\right]=\left[\begin{array}{cc} \pi_{0} & \pi_{1} \end{array}\right],$$

As a result, a set of linear equations was obtained:

(39) $$\{\begin{array}{c} p_{00}\pi_{0}+p_{10}\pi_{1}=\pi_{0}\\ p_{01}\pi_{0}+p_{11}\pi_{1}=\pi_{1} \end{array}$$

Under the following assumption:

(40) $$\pi_{0}+\pi_{1}=1$$

On solving the set of equations the limit probabilities π_0_, π_1_ were obtained.

Detailed information concerning the integrity model were presented in [16,30].

### 2.3. Simulated Trajectories

The simulation method was used to conduct analyses of AIS vessel trajectories.

For this purpose, the DR method and the EKF method can be used to detect abnormal behavior of the AIS system and for monitoring the integrity of AIS data.

Based on simulated data, five simulated trajectories were selected. Detailed information on the simulated trajectories is included in Table 1.

**Figure 2:**
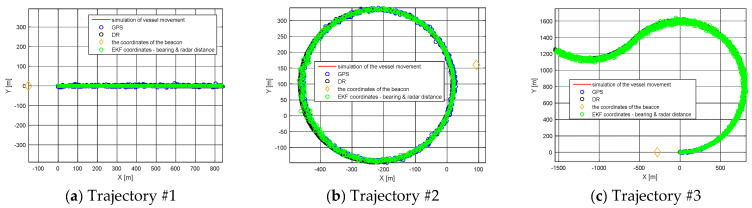
Simulation of the vessel movement and vessel trajectory estimations with the GPS, DR and RADAR EKF-SLAM method (**a**) Trajectory #1; (**b**) Trajectory #2; (**c**) Trajectory #3 [researchers’ elaboration].

The number of position iterations depended on the number of simulated position reports in individual simulated tracks. Trajectories present the data simulated from five different maneuvering vessels in various ways to check the possibilities of the algorithm estimating its coordinates. The speed of the ship during the simulation was 4.5 knots. The trajectories of the vessels are presented in Figure 2 in Section 3. Sea state 0, wind −1°B northward N.

## 3. Results

**Figure 3:**
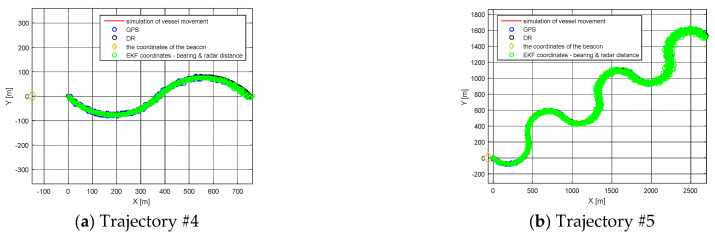
Simulation of the vessel movement and vessel trajectory estimations with the GPS, DR and RADAR EKF-SLAM method (**a**) Trajectory #4; (**b**) Trajectory #5 [researchers’ elaboration].

The results of the research tests are presented in Figure 2 and Figure 3 separately, according to the measurement trajectory number.

The integrity and accuracy of the results obtained in the ship’s motion model depend, inter alia, on the distance to navigational aid (beacon) and on the period time of the simulated trajectory.

**Figure 4:**
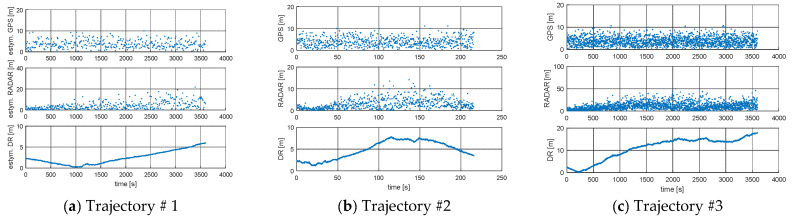
RMS of DR positions, RMS of RADAR positions, and RMS of GPS positions: (**a**) Trajectory #1; (**b**) Trajectory #2; (**c**) Trajectory #3 [researchers’ elaboration].

**Figure 5:**
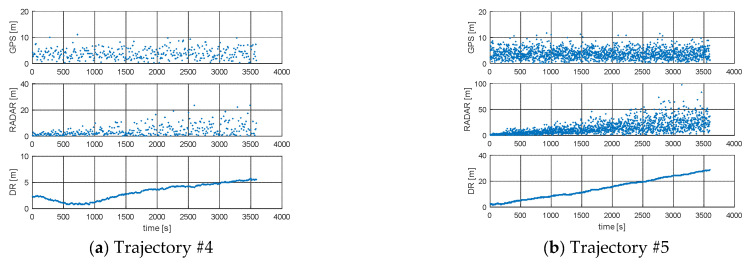
RMS of DR positions, RMS of RADAR EKF-SLAM positions, RMS of GPS positions: (**a**) Trajectory #4; (**b**) Trajectory #5 [researchers’ elaboration].

**Figure 6:**
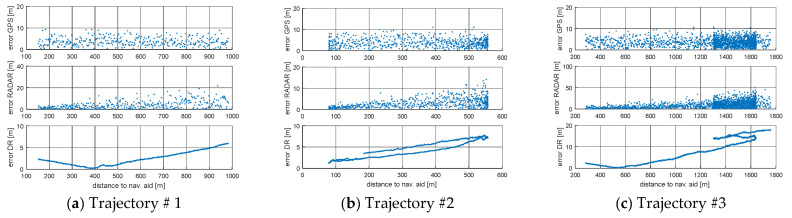
Error in determining the position of the ship depending on the distance from the navigation aid (beacon): (**a**) Trajectory #1; (**b**) Trajectory #2; (**c**) Trajectory #3.

**Figure 7:**
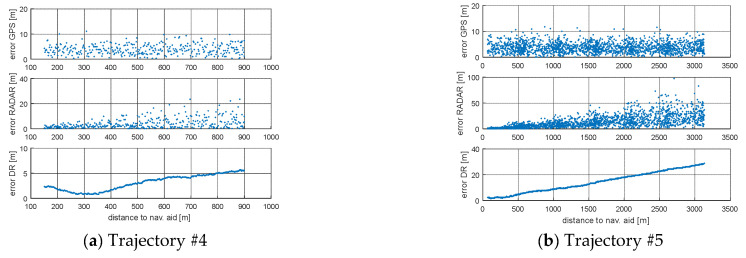
Error in determining the position of the ship depending on the distance from the navigation aid (beacon): (**a**) Trajectory #4; (**b**) Trajectory #5.

The Root Mean Square (RMS) for simulated positions and GPS, RADAR or DR positions are presented in Figure 4 and Figure 5 and Table 2. Figure 4 and Figure 5 present the error values of the ship’s position as a function of time. Figure 6 and Figure 7, on the other hand, show the position error values as a function of the distance from the navigation aid.

The accuracy of navigation solutions as a function of the interval between successive coordinates from AIS position reports depends on, among other factors, the distance to the navigation aid for the ship’s motion model using EKF-SLAM RADAR. In the case of a ship motion model based on GPS and DR simulations, the distance to the navigation aid does not matter. A slight increase in position error for the GPS model was observed when performing frequent turns (Trajectory #5). The DR model provided accurate estimation results for rectilinear motion (Trajectory #1) and short simulation time for circulation on the starboard side (Trajectory #4). When a ship circulates, the magnitude of the position error increases linearly as a function of time (Trajectory #2, Trajectory #3). The three ship traffic models can be used interchangeably depending on the type of navigation carried out and the availability of the GPS service. Choosing the right model is facilitated by Table 2, where RMS results for three models of ship movement are presented. The long distance to the navigation aid and the long return time (Trajectory #3) cause a linear increase in RMS as a function of time. In this situation, the best solution is to use a GPS model with an RMS error value, which arrives according to the normal distribution or on a specific road section which is appropriate to one of the three models, in order to minimize RMS.

The integrity model uses the data stream generated by the Polish Naval Academy’s ship simulator.

The input data to determine the reliability state of the AIS system in the Markov process are the recorded data from the simulating devices, namely:

- ship position—GPS receiver;
- bearing and distance—Navigation radar.

The DR model is based on the equations of motion known from kinematics. Data from devices were filtered with the intervals of 2, 3.33, and 10 s. It is possible to regulate the interval of the messages sent.

The ship moves along a trajectory planned in a device simulating motion. The simulator is a certified device, approved for training crews of vessels in accordance with the International Convention on Standards of Training, Certification and Watchkeeping for Seafarers (STCW).

The integrity of the simulated data from five ship motion trajectories was determined on the basis of the calculated RMS for three ship motion models. It was assumed that the integrity of AIS information could be maintained using one of the three ship motion models. Each of the models generates the coordinates of the object while moving along a given trajectory. For the radar system, the EKF-SLAM model was used to estimate the coordinates. For the GPS model, the position data was simulated by a GPS receiver. Additionally, data from AIS were recorded in AIVDO lines with message no. 1—Position report. These data coincided with the positions of the GPS receiver in accordance with the interval of messages sent by the AIS receiver. Time delays of AIS data in relation to GPS position did not take place due to the simulator data used. The data from the simulator was saved to txt files in order to determine the RMS. The determined mean square errors of the position were the basis for the development of the operational state criterion in Markov processes. In the quantization process of the system state, exceeding the threefold value of the square error (3*M_xy_*) meant a transition from the correct operation state to the system failure state.

The matrix of transition probabilities of the Markov chain was chosen based on the transition to system integrity for each state; thus, according to integrity model assumptions, the matrix of transitions probabilities of the Markov chain was created and is presented in Table 3.

**Figure 8:**
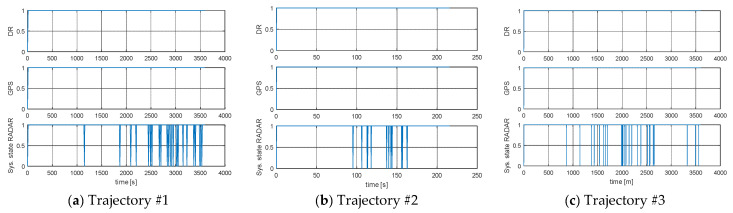
Intensity of transitions between states of the process: (**a**) Trajectory #1; (**b**) Trajectory #2; (**c**) Trajectory #3 [researcher’ elaboration].

**Figure 9:**
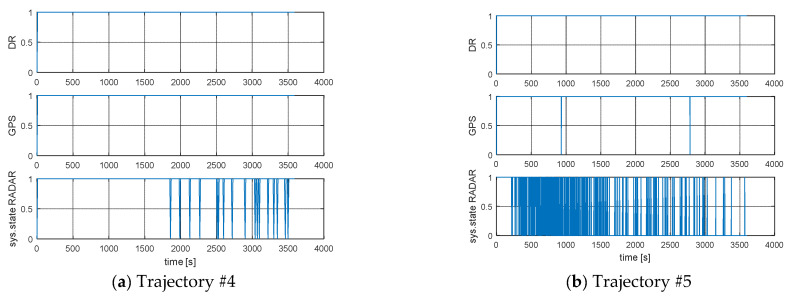
Intensity of transitions between states of the process: (**a**) Trajectory #4; (**b**) Trajectory #5; [researcher’ elaboration].

The detailed distribution of transitions to system integrity for each state is presented in Table 3 and Figure 8 and Figure 9.

The results show a high level of system integrity for five simulated trajectories. According to the integrity model, the use of the DR and GPS ship movement model, according to Table 3, gives similar results in the intensity of transitions between states. In the case of the EKF-Radar model, whose accuracy depends on the distance to the navigation aid, the intensity of transitions to a failure state (Trajectory #5). In other words, in 1331 cases out of 1800, the model returned a position accuracy of less than 3*M_xy_* (triple the value of the mean square error of the position depending on the movement model used by the ship).

A graph of the intensity of the transitions between different states of the process is presented in Figure 8 and Figure 9. Through quantization, the binary value corresponding to the system state was assigned to each of the integrity outcomes, namely:

S(1)=0—state of failure,

S(2)=1—working state.

On solving the set of Equations (37)–(40) limit probabilities π_0_, π_1_ were obtained and presented in Table 4.

Figure 8 and Figure 9 are graphical presentations of the intensity of transitions between the integrity states of the system depending on the model of ship movement used. These figures reflect the data in Table 3. The limit value of integrity characterizes the probability with which the system will be in a state of correct operation with the assumed model of ship movement. Significantly different results from the DR and GPS model compared to the RADAR model were observed for Trajectory #5 (Table 4). This was due to the time of registration and the distance from the navigation aid, as well as the large number of returns made by the ship.

## 4. Discussion

The integrity test of the AIS system based on the Markov process, discrete in states and in time, proved the high level of reliability of the dynamic data from the reference system. The limit probability of the system in operation was about 99% for both the GPS and DR traffic models. Significant reliability limitations were observed for the EKF-RADAR model, where the accuracy of the position depends on the bearing and distance to the navigation aid. In the EKF-RADAR model, the position error values were linear. Based on the assumptions for the estimation of the ship’s position and the assumptions made in the methodology of the AIS data integrity testing, only 1% of the time was the system in a failure state with the GPS and DR motion model. The state of failure concerned the moments when the accuracy exceeded 3*M_xy_* (triple the value of the mean square error of the position depending on the ship’s movement model being used). The similarity of the GPS and DR results was due to simulation errors being used in the calculations, which are slightly noisy, as well as the previously estimated coordinates by the GPS receiver, which is coupled with the AIS receiver. Simulations of position reports from five trajectories were made at the same time and in the same area—Gdańsk Bay. In such situations, the use of nonlinear methods of estimating a ship’s coordinates during circulation and changes of the course becomes important. The methodology of the DR model was used in [31], where a method was designed to restore the trajectory of an inland waterway ship based on the Automatic Identification System (AIS) data. The trajectory can be considered as a sequence of *n* points with temporal attribute timestamp (*t*) and positional attributes latitude (LAT) and longitude (LON). There are multiple methods for calculating similarities between the trajectories, with the most widely used metrics being based on Euclidean distance [32], (LCSS) Longest common subsequence [33], Hausdorff distance, Frechet distance, and Dynamic Time Warping (DTW) [34,35].

## 5. Conclusions

In this article, the integrity of dynamic data has been investigated using discrete-state and time-stochastic processes, i.e., Markov chains. The basis for the analysis of the integrity of dynamic AIS data in five simulated trajectories was the comparison of the position of ships from ideal simulated trajectory with the data obtained by using estimating functions from three models of ship movement.

The research outcomes of the five simulated trajectories were presented to demonstrate the abilities of three motion models, namely, EKF-SLAM, GPS and DR, to detect limitations in AIS system dynamic data integrity.

The results of GPS, EKF-SLAM and DR motion models were compared with five ideal trajectories. The comparison outcomes were used for the integrity assessment of the simulated AIS data. For failure detection, the discrete-time, discrete-state Markov Process, namely, a Markov chain, was implemented. The test scenarios comprised simulated data from five simulated trajectories. It was confirmed that three process models returned differential investigation outcomes. The models were checked in turning maneuvers and straight-line motion. The Markov Process proved to be successful in the detection of severe discrepancies between the model outputs and ideal simulated data. It was also precise in isolating the time of failure. Only a small number of failures with regard to data integrity were observed for DR and GPS model. The results of the research have proven the high level (~99%) of integrity of the dynamic information of the AIS system. Only for the EKF-SLAM model, where the measurement vector consisted of bearing and radar distance, was the integrity factor at a level of 93~98% for trajectories #1–4, and only 26% for trajectory #5. Such a large discrepancy in the outcomes was the result of frequent changes in course and the constantly increasing distance to the navigation aid. The research hypothesis was proven. Both RADAR-EKF and DR estimating ship coordinates can serve as tools for simulated reconstruction of the ship trajectory, which is a basis for the verification of the AIS system integrity using Markov-chain-based stochastic process. The use of the GPS, DR and RADAR-EKF models presented in the paper will be limited by the simulated data of the AIS system.

The added value of the proposed method of estimating coordinates in the RADAR-EKF model is the determination of bearing and distance to navigational aid and the use of random errors for the computation of the control vector in predicting a ship’s state. On the other hand, Markov processes are one of the best models for estimating the integrity, availability and reliability of devices. They present the intensity of the system being in a state of failure or other state on the basis of initial assumptions relying on a defined set of system states. The method that uses the set of operational states in the quantization process with the possibility of comparing the results is the hypothesis testing method, presented at the beginning of the paper [10], using the Chi-square distribution, and the GLR model, mentioned in the introduction to this work. In the methods presented in [10], it was established that the system can exist in two states in terms of reliability. A binary presentation of the system state in terms of reliability in this method was used to assess the degree of system operation reliability, giving similar test results based on the presented figures of the intensity of transitions between the states of the system [10]. The much simpler method based on the Chi-square test gave similar results of the intensity of transitions between states and the detection of operational failure of the system compared to the more advanced model based on GLR test hypotheses [10].

One direction of future research on the reliability of dynamic information in the AIS system is the application of a ship movement model with the use of a particle filter. This would provide excellent estimates for highly noisy measurements. The use of particle filters and discrete in state and time Markov processes may result in more accurate results in AIS reliability tests. Additionally, the measurement vector in the ship motion model should take into account data from the synthetic aperture radar or inertial navigational system.

## Figures and Tables

**Table 1:** Vessel movement trajectory simulations [researchers’ elaboration].

	Trajectory #1	Trajectory #2	Trajectory #3	Trajectory #4	Trajectory #5
initial COG [deg]	000	066	000	315	315
ROT [deg/min]	000	010	005	003	015
Turn after a time of [min]	0	2	45	30	10
SOG [knots]	4.5	4.5	4.5	4.5	4.5
Δt [s]	10	3.33	2	10	2
simulation time [s]	3600	2160	3600	3600	3600
σ(GPS) [m]	3	3	3	3	3
(β) [deg]	0.3	0.3	0.3	0.3	0.3
(θ) [deg]	1.5	1.5	1.5	1.5	1.5
initial dist. to nav. aid [m]	150	85	280	150	63

**Table 2:** Root mean square error for DR, RADAR and GPS model [researchers’ elaboration].

Campaign Number	M(GPS) xy [m]	M(RADAR) xy [m]	M(DR) xy [m]
Trajectory #1	3.8	4.3	2.5
Trajectory #2	3.9	3.1	4.9
Trajectory #3	3.7	11.0	11.0
Trajectory #4	3.9	4.3	3.2
Trajectory #5	3.8	14.4	14.5

**Table 3:** The intensity of transitions from the state i ∈ S to the state j ∈ S in the Markov chain [researchers’ elaboration].

Process Model	Trajectory No.	S(0)→S(0)	S(0)→S(1)	S(1)→S(0)	S(1)→S(1)	Number of States
DR	1	0	1	0	359	360
GPS	1	0	1	0	359	360
RADAR	1	3	22	21	314	360
DR	2	0	1	0	647	648
GPS	2	0	1	0	647	648
RADAR	2	1	13	12	622	648
DR	3	0	1	0	1799	1800
GPS	3	0	1	0	1799	1800
RADAR	3	1	27	26	1746	1800
DR	4	0	1	0	359	360
GPS	4	0	1	0	359	360
RADAR	4	0	18	17	325	360
DR	5	0	1	0	1799	1800
GPS	5	0	3	2	1795	1800
RADAR	5	1140	191	191	278	1800

**Table 4:** The limit probability of failure state $\pi_{0}$ and working state $\pi_{1}$ for GPS, RADAR and DR estimation method [researchers’ elaboration].

Process Model	DR	GPS	RADAR
trajectory #1	$\pi_{0}$ $=0.003,\;\pi_{1}=0.997$	$\pi_{0}$ $=0.003,\;\pi_{1}=0.997$	$\pi_{0}$ $=0.069,\;\pi_{1}=0.931$
trajectory #2	$\pi_{0}$ $=0.002,\;\pi_{1}=0.998$	$\pi_{0}$ $=0.002,\;\pi_{1}=0.998$	$\pi_{0}$ $=0.021,\;\pi_{1}=0.978$
trajectory #3	$\pi_{0}$ $=0.001,\;\pi_{1}=0.999$	$\pi_{0}$ $=0.001,\;\pi_{1}=0.999$	$\pi_{0}$ $=0.016,\;\pi_{1}=0.984$
trajectory #4	$\pi_{0}$ $=0.003,\;\pi_{1}=0.997$	$\pi_{0}$ $=0.003,\;\pi_{1}=0.997$	$\pi_{0}$ $=0.050,\;\pi_{1}=0.950$
trajectory #5	$\pi_{0}$ $=0.001,\;\pi_{1}=0.999$	$\pi_{0}$ $=0.002,\;\pi_{1}=0.998$	$\pi_{0}$ $=0.739,\;\pi_{1}=0.261$

## Data Availability

Not applicable.

## References

[1] Specht C. (2021). Radio navigation systems: Definitions and classifications. J. Navig..

[2] Jaskólski K. (2017). Automatic Identification System (AIS) dynamic data estimation based on discrete Kalman Filter (KF) algorithm. Zesz. Nauk. Akad. Mar. Wojennej.

[3] Jaskólski K. (2014). The Availability of Automatic Identification System (AIS) Based on Latency Position Reports in The Gulf of Gdansk. Annu. Navig..

[4] Specht C., Rudnicki J. (2016). A Method for The Assessing of Reliability Characteristics Relevant to an Assumed Position-Fixing Accuracy in Navigational Positioning Systems. Polish Marit. Res..

[5] Specht M. (2019). Method of Evaluating the Positioning System Capability for Complying with the Minimum Accuracy Requirements for the International Hydrographic Organization Orders. Sensors.

[6] Felski A., Jaskólski K., Banyś P. (2015). Comprehensive Assessment of Automatic Identification System (AIS) Data Application to Anti-collision Manoeuvring. J. Navig..

[7] Fossen S., Fossen T.I. Extended Kalman Filter Design and Motion Prediction of Ships Using Live Automatic Identification System (AIS) Data. Proceedings of the 2018 2nd European Conference on Electrical Engineering and Computer Science (EECS).

[8] Kaniewski P. (2010). Structures, Models and Algorithms in Integrated Positioning and Navigation Systems (Struktury, Modele i Algorytmy w Zintegrowanych Systemach Pozycjonujących i Nawigacyjnych).

[9] Särkkä S., Tamminen T., Vehtari A., Lampinen J. (2004). Probabilistic Methods in Multiple Target. Tracking—Review and Bibliography.

[10] Siegert G., Banys P., Martinez C.S., Heymann F. EKF based trajectory tracking and integrity monitoring of AIS data. Proceedings of the 2016 IEEE/ION Position, Location and Navigation Symposium (PLANS).

[11] Konatowski S., Kaniewski P., Matuszewski J. (2016). Comparison of Estimation Accuracy of EKF, UKF and PF Filters. Annu. Navig..

[12] Xu T., Liu X., Yang X. Ship Trajectory Online Prediction Based on BP Neural Network Algorithm. Proceedings of the 2011 International Conference of Information Technology, Computer Engineering and Management Sciences.

[13] Guo S., Zhang X., Zheng Y., Du Y. (2020). An Autonomous Path Planning Model for Unmanned Ships Based on Deep Reinforcement Learning. Sensors.

[14] Yang F., Qiao Y., Wei W., Wang X., Wan D., Damaševičius R., Woźniak M. (2020). DDTree: A Hybrid Deep Learning Model for Real-Time Waterway Depth Prediction and Smart Navigation. Appl. Sci..

[15] Jakubowski B. (2003). Niezawodnościowe Aspekty Przetwarzania Informacji w Zintegrowanym Systemie Nawi-Gacyjnym (Reliability-Based Aspects of Data Processing in an Integrated Navigational System).

[16] Jaskólski K. (2014). Availability and Integrity Model of Automatic Identification System (AIS) Information.

[17] Chang Y., Chen C. (2020). A Kullback-Leibler information control chart for linear profiles monitoring. Qual. Reliab. Eng. Int..

[18] Abba S.I., Gaya M.S., Yakubu M.L., Zango M.U., Abdulkadir R.A., Saleh M.A., Hamza A.N., Abubakar U., Tukur A.I., Wahab N.A. Modelling of Uncertain System: A comparison study of Linear and Non-Linear Approaches. Proceedings of the 2019 IEEE International Conference on Automatic Control and Intelligent Systems (I2CACIS).

[19] Harati-Mokhtari A., Wall A., Brooks P., Wang J. (2007). Automatic Identification System (AIS): Data Reliability and Human Error Implications. J. Navig..

[20] Felski A., Jaskolski K. (2013). The Integrity of Information Received by Means of AIS During Anti-collision Manoeuvring. TransNav Int. J. Mar. Navig. Saf. Sea Transp..

[21] Lensu M., Goerlandt F. (2019). Big maritime data for the Baltic Sea with a focus on the winter navigation system. Mar. Policy.

[22] Fournier M., Casey Hilliard R., Rezaee S., Pelot R. (2018). Past, present, and future of the satellite-based automatic identification system: Areas of applications (2004–2016). WMU J. Marit. Aff..

[23] Yang D., Wu L., Wang S., Jia H., Li K.X. (2019). How big data enriches maritime research—A critical review of Automatic Identification System (AIS) data applications. Transp. Rev..

[24] Wang J., Zhu C., Zhou Y., Zhang W. (2017). Vessel Spatio-temporal Knowledge Discovery with AIS Trajectories Using Co-clustering. J. Navig..

[25] Alizadeh D., Alesheikh A.A., Sharif M. (2021). Vessel Trajectory Prediction Using Historical Automatic Identification System Data. J. Navig..

[26] Sun Y., Chen X., Jun L., Zhao J., Hu Q., Fang X., Yan Y. (2021). Ship trajectory cleansing and prediction with historical ais data using an ensemble ann framework. Int. J. Innov. Comput. Inf. Control..

[27] International Telecommunication Union (2014). Technical Characteristics for an Automatic Identification System Using Time Division Multiple Access in the VHF Maritime Mobile Band (Recommendation ITU-R M.1371-4).

[28] Dąbrowski P.S., Specht C., Felski A., Koc W., Wilk A., Czaplewski K., Karwowski K., Jaskólski K., Specht M., Chrostowski P. (2019). The Accuracy of a Marine Satellite Compass under Terrestrial Urban Conditions. J. Mar. Sci. Eng..

[29] Marchel Ł., Naus K., Specht M. (2020). Optimisation of the Position of Navigational Aids for the Purposes of SLAM technology for Accuracy of Vessel Positioning. J. Navig..

[30] Jaskólski K. (2013). Zastosowanie łańcuchów Markowa w badaniu potencjalnych możliwości wykorzystania informacji automatycznego systemu identyfikacji (AIS) w aplikacjach antykolizyjnych. Zesz. Nauk. Akad. Mar. Wojennej.

[31] Sang L., Wall A., Mao Z., Yan X., Wang J. (2015). A novel method for restoring the trajectory of the inland waterway ship by using AIS data. Ocean. Eng..

[32] Jonker R., De Leve G., Van Der Velde J.A., Volgenant A. (1980). Technical Note—Rounding Symmetric Traveling Salesman Problems with an Asymmetric Assignment Problem. Oper. Res..

[33] Kearney J.K., Hansen S. (1990). Stream Editing for Animation.

[34] Sakoe H., Chiba S. (1978). Dynamic programming algorithm optimization for spoken word recognition. IEEE Trans. Acoust..

[35] Soong F.K., Rosenberg A.E. (1988). On the use of instantaneous and transitional spectral information in speaker recognition. IEEE Trans. Acoust..

